# Overcoming Immune Deficiency with Allogrooming

**DOI:** 10.3390/insects14020128

**Published:** 2023-01-26

**Authors:** Mark S. Bulmer, Bruno A. Franco, Aditi Biswas, Samantha F. Greenbaum

**Affiliations:** Department of Biological Sciences, Towson University, 341 Smith Hall, 8000 York Rd., Towson, MD 21252, USA

**Keywords:** social immunity, innate immunity, entomopathogens, alarm behavior, termicins

## Abstract

**Simple Summary:**

We investigated whether two closely related termite species differ in their reliance on social or individual immunity against two species of pathogenic fungi, which were all collected from the same location. Our results indicate that mutual grooming (allogrooming) is highly effective at limiting fatal infections to the extent that it can compensate for relatively weak individual immune defenses. After pathogens are encountered, termites use an alternate strategy for social distancing and limiting the spread of contagious disease and instead come together to clean each other. Variation in the intensity of this behavioral response indicates a nuanced response to the level of threat.

**Abstract:**

Allogrooming appears to be essential in many social animals for protection from routine exposure to parasites. In social insects, it appears to be critical for the removal of pathogenic propagules from the cuticle before they can start an infectious cycle. For subterranean termites, this includes fungal spores commonly encountered in the soil, such as *Metarhizium* conidia, that can quickly germinate and penetrate the cuticle. We investigated whether there is a difference in reliance on social and innate immunity in two closely related subterranean termites for protection from fatal infections by two locally encountered *Metarhizium* species. Our results indicate that relatively weak innate immunity in one termite species is compensated by more sustained allogrooming. This includes enhanced allogrooming in response to concentrations of conidia that reflect more routine contamination of the cuticle as well as to heavy cuticular contamination that elicits a networked emergency response.

## 1. Introduction

Social mechanisms of defense can be highly effective at counteracting susceptibility to contagious disease despite sociability favoring its spread [1,2]. In termites and ants, the evolution of effective social mechanisms of immune defense appears to have relaxed selective pressure on individual innate immune mechanisms [3,4,5,6]. After pathogens are encountered, the threat of disease in social insects will depend on the efficacy of innate frontline defenses, which include physical barriers to infection. Many fungal pathogens produce asexual spores (conidia) that can directly penetrate the insect cuticle. Social immune defenses that help protect the cuticle, including allogrooming and secreted antifungals, appear to be especially important in termites for defense against *Metarhizium* spp [7,8,9]. *Metarhizium* conidia are ubiquitous in the soil that many termites move through while foraging for food and reach concentrations that sustain a constant threat of infection [10]. 

Foraging termites, such as species of the genus *Reticulitermes* move through soil to expand their colonies’ range and access multiple feeding sites, which are usually wood resources resting on the soil surface [11]. Their colonies’ subterranean networks can extend over tens of meters, contain over a hundred thousand individuals and reach densities of over a hundred colonies per hectare [12]. They evolved from an ancestor with colonies restricted to a single piece of wood [13,14]. This transition would have resulted in increased encounters with predators, pathogens, and competitors and coincided with the evolution of a dedicated true worker caste that usually foregoes direct reproduction. Improved hygiene is likely to have been critical for this transition and the subsequent radiation and current ecological success of termites, whose contribution to wood decay can exceed microbial decomposition [15,16] and who appear poised to rapidly expand globally as temperatures rise [17]. During the transition to foraging, a relaxation of selective pressure on innate immune genes coincided with its intensification on two genes that produce secreted antifungal proteins [6]. These proteins appear to be essential for the removal of fungal conidia with allogrooming. The contrasting selective pressure on components of individual innate and social mechanisms of defense in the past suggests that there continues to be an adaptive tradeoff in termites’ reliance on individual innate and social immunity for protection from fungal disease. This is supported by the observation that innate immune gene expression is lower in groups than individuals of *Reticulitermes* workers that have been exposed to *Metarhizium* conidia [18]. Allogrooming reduces the need to activate internal immune systems.

In this study, we investigated differences in social and innate immunity in two species of subterranean termites against two local species of *Metarhizium* that encounter each other naturally. The *Metarhizium* species were isolated from the soil underneath or next to logs in which termites were collected. The two termite species, *Reticulitermes virginicus* and *Reticulitermes flavipes* were collected from the same location in Maryland. Although they appear to be agonistic, they are often found foraging next to each other or even occasionally in the same log. Interspecific aggression between workers frequently results in fatal wounds in Petri dish agonism assays [19]. The antifungal activity of cuticular washes of *R. virginicus* workers has previously been shown to be more effective at inactivating *Metarhizium* conidia than washes from *R. flavipes* workers [20]. This suggests that although these two termites are closely related and encounter the same pathogens, they may differ in their reliance on individual innate or social immunity. We used a comparative approach to investigate whether there is a difference between the reliance on and integration of social and innate immunity and whether there is a potential tradeoff between the two. Enhanced social immunity may be associated with less effective innate immune mechanisms, which may include the potency of secreted antifungals, and vice versa. Weaker individual immunity may be compensated by more effective social immunity.

The survivorship of late instar termite workers in groups of twelve, pairs, or singletons was investigated after they had been challenged with fungi from either *Metarhizium robertsii* or *Metarhizium brunneum*. Groups of 12 workers were briefly exposed to a high dose of the fungus. This potentially fatal concentration causes similar behavioral responses to that observed when termites encounter a sporulating cadaver, in some cases eliciting a substantial increase in an alarm response that results in rapid longitudinal oscillatory movements, LOMs [21,22,23]. The frequency of LOMs in the first 12 min after termites are exposed to conidia of different strains and species of *Metarhizium* is positively correlated with the frequency of bouts of allogrooming. LOMs, which are easier to measure than allogrooming, can therefore be used as an indirect measure of allogrooming levels. Detection of the fungus appears to be delayed until the surfaces of termites are contaminated with conidia, which suggests that recognition receptors associated with mouthparts are critical for the onset of elevated alarm and allogrooming.

Pairs and singletons were exposed to a low dose of the fungus that is more likely to reflect the levels of exposure that termites encounter when they construct galleries through the soil, in contrast to direct encounters with sporulating cadavers. Unlike single termites, termite pairs are almost completely resistant to fatal infections at this dose over 10 days. To tease apart social and innate immunity at this low dose, pairs of termites were separated at different time points shortly after challenges with fungal conidia, and the survivorship of these pairs was compared to singletons that had no opportunities for social interactions after fungal challenges. Allogrooming and LOMs were observed before the separation time points of termite pairs. In addition, the antifungal activity of crude extracts of whole termites was tested to further assess differences in innate immunity between the two termite species in response to the two species of fungi.

## 2. Methods

### 2.1. Study Organisms

The termites *R. flavipes* and *R. virginicus*, and the fungi *M. brunneum* and *M. robertsii*, were collected from secondary growth deciduous forest in Baltimore County (Pleasant Hills and Monkton), Maryland. Termite species were identified using intra- and interspecific agonism assays [19] and mitochondrial rRNA sequences [4]. The *Metarhizium* species were isolated from soil samples near termite collection sites (<30 cm) using a baiting technique with *Tenebrio molitor* larvae [24]. The conidia and mycelia of two clonal lines of the species were used for DNA purification with a QIAGEN DNeasy Blood and Tissue kit. The 5′ region of translation elongation factor 1-alpha was PCR-amplified using PCR primers EF1T and EF2T [25]. These regions were Sanger-sequenced (Psomagen, Rockville, MD) for species identification. Foraging groups of *R. flavipes* and *R. virginicus* (>1000) and the wood in which they were feeding were collected and stored in plastic containers under dark, moist conditions at 25 °C. Termites belonging to the same species were collected from sites that were separated by at least 60 m, which is usually sufficient to ensure that collections are from different colonies [12]. These separate collections are referred to as colonies below. Termites were collected in the summer of 2018 and 2019 for the experiments with the groups of 12 as well as in vitro experiments with termite crude extracts, and 2021 and 2022 for the experiments with pairs and singletons. Termites were used in their respective experiments within 4 months of collection. Workers were gently removed from wood pieces and maintained in groups of 12 in 55 mm diameter Petri dishes lined with moistened filter paper (Whatman 1) for 24 h in ambient light prior to being used for our experiments. This allowed them to acclimatize to light and clean their cuticular surfaces of particulate debris prior to the fungal exposures. We did not include soldiers as they do not groom nestmates, represent 1–2% of the colony, and are not consistently found in foraging groups.

### 2.2. Survivorship and Alarm Response for Groups of Twelve

Groups of 12 *R. flavipes* or *R. virginicus* workers were exposed to a high dose of 10^6^ conidia mL^−1^ of 0.05% Tween 80 (MilliporeSigma, Burlington, MA, USA) or 0.05% Tween 80 for controls. Tween 80 was included to help break up clumps of conidia when preparing the suspensions. The day before these treatments, all 12 termites were marked with a small dot of colored enamel paint on the dorsal side of their abdomens so that they could be individually identified. Allogrooming did remove the paint from a few individuals, but these individuals could still be individually identified at the time of death by surveying the survivors and by natural individual variation in the workers, such as the degree of dark coloration in the abdomen. The termites used for each replicate of 12 challenged termites and controls were from different colonies. Five colonies were used for both *R. flavipes* and *R. virginicus* (n = 480 workers). Experiments with *M. robertsii* colonies were repeated after three months to test for consistency in survivorship. In total, there were 60 replicates of 12 or 11 termites (n = 709). In 11 replicates a termite died prior to fungal exposure, which appeared to be due to handling when paint marking. Termites were exposed to conidia prepared from a clonal culture of either *M. robertsii* or *M. brunneum.* Workers were added to 1.5 mL tubes containing 500 μL of the conidia dilutions, or 0.05% Tween 80 for controls, with a small plastic funnel and gently agitated for 10 s to ensure complete immersion. The workers were then deposited onto filter paper to absorb excess fluid, transferred to 55 mm diameter Petri dishes lined with moistened filter paper (Whatman 1), and video recorded for 12 min. Mortality was monitored daily for two weeks. Dead individuals were removed, surface sterilized with 70% ethanol, and placed on moist filter paper in Petri dishes (55 mm) maintained at 24 °C and 100% humidity for confirmation of *Metarhizium* infection (green muscardine growth after 3–4 days). The total number of LOMs observed in the videos over 12 min were scored for each individual termite. LOMs lasted between 2–7 s and had to have stopped and then resumed to be scored as separate events [23]. 

### 2.3. Survivorship and Allogrooming for Pairs and Singletons

The survivorship of termite pairs was compared to singletons after exposure to a low dose (10^4^ conidia mL^−1^) of conidia that did not cause excessive mortality and likely reflects concentrations that termites encounter when moving through soil [10]. Pairs and singletons were compared to measure survival afforded by social mechanisms of defense. Groups of 12 workers were initially challenged with or without fungi as described above, except that conidial suspensions of approximately 10^7^ conidia mL^−1^ were prepared in 0.1% Tween 80 and vigorously vortexed prior to dilutions with sterile water to 10^4^ conidia mL^−1^. Tween 80 was not included in the dilutions because suspensions of individual conidia could be maintained at low concentrations and because the surfactant may interfere with adhesion of conidia to the cuticle. These individuals were not paint marked. Immediately after treatments and before termites had the opportunity to interact, they were divided into pairs or singletons in the wells of 24-well plastic cell culture plates (CELLTREAT Scientific, Pepperell, MA). Each well was lined with moistened filter paper (Whatman 1). For each termite/fungal combination (n = 4), four termite pairs were separated into singletons after 15 min, 2 h, 5.5 h, 8 h, 27 h (n = 160), and for each termite/fungal combination 12 termites that never had the opportunity to interact were separated into fungal treated or control singletons (n = 96). These individual termites were also divided into 24-well plates lined with filter paper. The mortality of the termites was subsequently monitored over ten days, and dead individuals were removed for confirmation of infection by *Metarhizium*. A single colony was used for both *R. flavipes* treated with *M. robertsii* or *M. brunneum* and *R. virginicus* treated with *M. robertsii* or *M. brunneum* (n = 256). Six untreated pairs were also maintained in 24-well plates for 10 days for each species.

A separate experiment with the same colonies focused on recording allogrooming prior to the separation times of the pairs at 15 min, 2 h, 5.5 h, 8 h, and 27 h. Six different pairs of each termite species were treated with each fungus or water for controls and scored for allogrooming for 15 min before the separation time points (n = 180). Allogrooming events were scored when mouth-to-body contact between two termites involved the movement of mouthparts (palps), and the duration of each uninterrupted allogrooming event was recorded. Allogrooming was scored by a single observer who was blind to which treatment termites had received. LOMs were also scored but were negligible and not used in the analysis. Self-grooming was not observed. 

### 2.4. In Vitro Antifungal Activity

Twelve workers from 5 *R. flavipes* or *R. virginicus* colonies were cold immobilized in 1.5 mL Eppendorf tubes, and 120 μL of cold, sterile water was added to each tube. The workers from both species used in this study were approximately the same size (length 4–5 mm). The termites were homogenized with a plastic pestle, and the extract was centrifuged at 16,000 g for 2 min. The supernatant was filter sterilized with 0.22 μm Ultrafree filters (MilliporeSigma, Burlington, MA, USA). Ten μL of the filtrate was incubated for 24 h with ten μL containing 100 conidia of *M. robertsii* or *M. brunneum* and 20 μL of 100 μg mL^−1^ ampicillin to further avoid bacterial contamination. Controls included 10 μL of sterile water in place of the crude extract. Two replicates for each incubated mix were spread on potato dextrose agar (PDA) supplemented with 50 μg mL^−1^ ampicillin (60 mm Petri dishes) and incubated at RT in the dark for 4–5 days. Antifungal activity was measured by comparing colony-forming units (CFUs) in crude extract treatments to controls. 

### 2.5. Statistics

Kaplan–Meier estimates of mean survival times were used for pairwise comparisons of survivorship. Hazard ratios of death (HRs) were calculated with Cox regression analysis. We used Cox regression to investigate differences in mortality between individuals exhibiting LOMs or not exhibiting LOMs while controlling for other categorical variables (fungal treatment or control, species of fungi, species of termite, colony of origin). LOMs were categorized as absent or present in individuals because of the frequency of outliers and lack of normality. In the survival analysis with termite pairs and singletons, the HR was based on a comparison between conidia-treated pairs (separated at different time points) and conidia-treated singletons. These HRs, therefore, provide a measure of survival afforded by social mechanisms of defense while controlling for individual mechanisms of defense. The association between time spent as a pair and change in HR was measured with Pearson correlations. The distribution of LOMs among different treatments were compared with a Kruskal–Wallis test with Bonferroni corrections for multiple testing for pairwise comparisons. Mean CFUs or duration of allogrooming events were compared with an ANOVA test with Bonferroni corrections for pairwise comparisons. We used IBM SPSS Statistics (version 28) for the statistical tests.

## 3. Results

### 3.1. Survivorship and Alarm of Challenged Groups of Twelve

The Cox regression model of survivorship that included categorical variables for fungal treatment, LOMs, termite species, fungi species, and colony of origin was significant (Chi-square = 246.29, df = 12, *p <* 0.001). Challenged termites were 9.28 times more likely to die than untreated termites (Wald = 143.05, *p <* 0.001), while controlling for the other variables. Termites that displayed LOMs (n = 422) were 1.52 times more likely to survive (Wald = 8.30, *p =* 0.004) than individuals that did not display LOMs (n = 286). *R. virginicus* workers were 2.08 times more likely to die than *R. flavipes* workers (Wald = 6.31, *p =* 0.012). Fungal species did not contribute significantly to the model (Wald = 0.841, *p =* 0.359). Colony of origin contributed significantly to the model (Wald = 50.16, *p <* 0.001), which is consistent with previously reported colony variation in *Reticulitermes* resistance to *Metarhizium* infections [24]. Survivorship did not differ between the same colony when the experiment was repeated 3 months later with workers that were exposed to *M. robertsii* (Breslow pairwise comparison, Chi-square = 0.342, *p =* 0.559).

**Figure 1:**
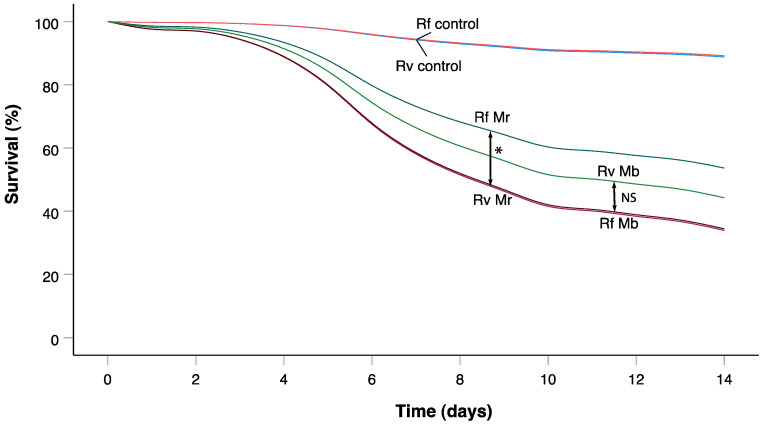
Survivorship for groups of 12. Rf corresponds with *R. flavipes*, Rv with *R. virginicus*, Mr with *M. robertsii* and Mb with *M. brunneum*. Significant survival differences for pairwise comparisons were based on Breslow tests (generalized Wilcoxon tests). * Significant difference between treatments, NS no significant difference between treatments (*p <* 0.025 Bonferroni adjusted α).

Most deaths in the challenged termites were attributable to *Metarhizium* infection (>90% confirmation). For challenged termites with *M. robertsii*, 129 deaths were observed among 237 individuals, and the mean survival time for *R. virginicus* (9.10 days) was significantly lower than for *R. flavipes* (10.98 days)(Breslow = 10.05, df = 1, *p =* 0.002) (Figure 1). For challenged termites with *M. brunneum*, 79 deaths were observed among 120 individuals, and the mean survival time for *R. flavipes* (9.70 days) was lower than for *R. virginicus* (10.68 days) but not significantly (Breslow = 2.74, df = 1, *p =* 0.098).

**Figure 2:**
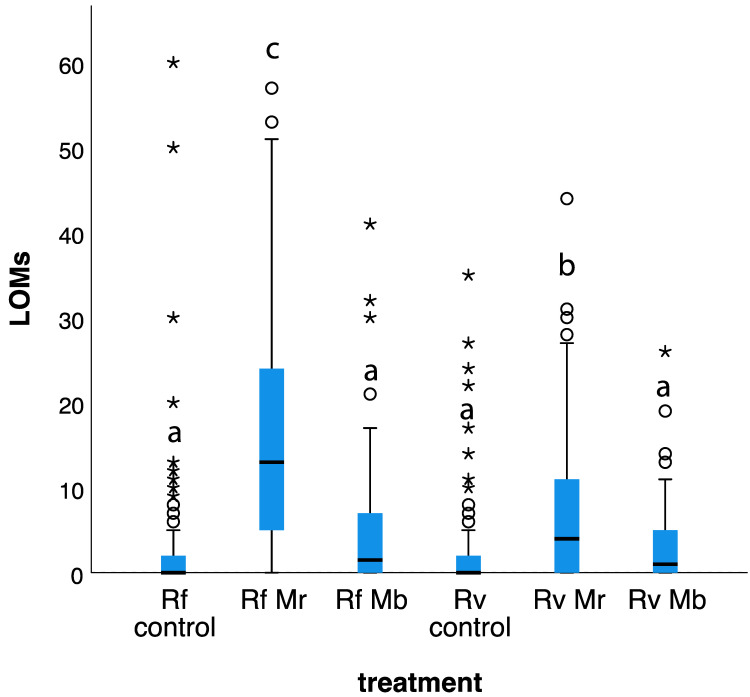
Alarm response to fungal challenge. Boxplots represent the distribution of LOMs (LOMs scored in 709 paint-marked individuals). Boxes correspond with the distance between upper and lower quartiles from the median (IQR). Whiskers correspond with 1.5 × IQR. Circles and asterisks depict outliers beyond 1.5 × IQR or 3 × IQR, respectively. Different letters indicate significant differences between distributions for fungal and control treatments (Bonferroni adjusted). Rf corresponds with *R. flavipes*, Rv with *R. virginicus*, Mr with *M. robertsii* and Mb with *M. brunneum*.

The distribution of LOMs (Figure 2) among fungal and control treatments was significantly different (Kruskal–Wallis = 176.897, df = 5, *p <* 0.001). The frequency of LOMs was not significantly different between *M. brunneum* treatments and their respective controls for *R. flavipes* (adjusted *p =* 0.097) and *R. virginicus* (adjusted *p =* 1.000). However, the frequency of LOMs was significantly greater for *M. robertsii* treatments compared to their respective controls for *R. flavipes* (adjusted *p <* 0.001) and *R. virginicus* (adjusted *p <* 0.001) (Figure 2). For the *M. robertsii* treatment, the frequency of *R. flavipes* LOMs was significantly greater than *R. virginicus* LOMs (adjusted *p <* 0.001). However, for the *M. brunneum* treatment, the frequency of *R. flavipes* LOMs was not significantly different from *R. virginicus* LOMs (adjusted *p =* 1.000). In contrast to *M. brunneum*, groups of 12 termites challenged with *M. robertsii* conidia resulted in a significant behavioral response. 

### 3.2. Survivorship of Challenged Pairs and Singletons

**Figure 3:**
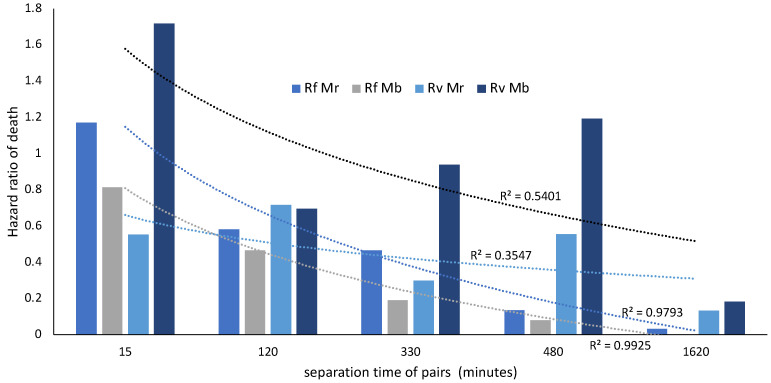
The survivorship of two termites versus one was compared with hazard ratios of death (n = 80 pairs, 96 singletons). Rf corresponds with *R. flavipes*, Rv with *R. virginicus*, Mr with *M. robertsii* and Mb with *M. brunneum*. Time was log transformed for calculation of the R^2^ values.

The survivorship of termite pairs separated at different times shortly after pathogen challenge was compared to the survivorship of challenged singletons with hazard ratios of death (HRs). An HR of 1 corresponds with equal survivorship in the separated pairs and singletons over ten days. Values less than one indicate that there is reduced mortality in the separated pairs compared to the singletons. The correlation between time spent as a pair and the reduction in HR was only significant for *R. flavipes* treated with either *M. robertsii* (r = −0.978, *p =* 0.004) or *M. brunneum* (r = −0.983, *p =* 0.003), and not for *R. virginicus* treated with either *M. robertsii* (r = −0.643, *p =* 0.242) or *M. brunneum* (r = −0.812, *p =* 0.095). This is also reflected in the R^2^ values from linear regressions shown in Figure 3 (time was log transformed for calculation of the Pearson correlations and R^2^ values). No death was observed in untreated pairs (6 pairs for each termite species) and one death in 16 treated pairs (4 pairs for each termite/fungi combination) that were never separated over 10 days.

**Figure 4:**
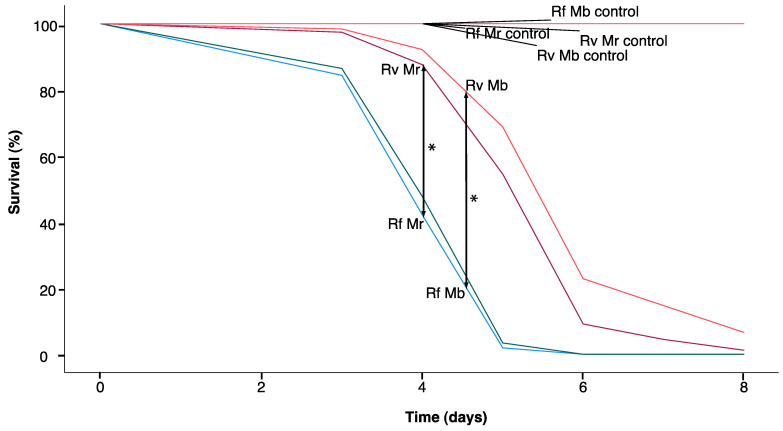
Survival of single termites after a fungal challenge (n = 96). Rf corresponds with *R. flavipes*, Rv with *R. virginicus*, Mr with *M. robertsii* and Mb with *M. brunneum*. See text for details on statistical differences between treatments. Significant survival differences for pairwise comparisons were based on Breslow tests (generalized Wilcoxon tests). * Significant difference between treatments (*p <* 0.025 Bonferroni adjusted α).

For the survivorship of pathogen-challenged single termites (Figure 4), 47 deaths were observed among 48 individuals, and the mean survival time for *R. flavipes* (4.21 days) was significantly lower than for *R. virginicus* (6.17 days)(Breslow, chi-square = 33.927, df = 1, *p <* 0.001). This relationship held for both species of fungi: For *M. robertsii* exposure, the mean survival time for *R. flavipes* (4.25 days) was significantly lower than for *R. virginicus* (5.83 days) (chi-square = 16.133, df = 1, *p <* 0.001); For *M. brunneum* exposure, the mean survival time for *R. flavipes* (4.17 days) was significantly lower than for *R. virginicus* (6.50 days) (chi-square = 20.994, df = 1, *p <* 0.001).

### 3.3. Antifungal Activity of Crude Extracts

**Figure 5:**
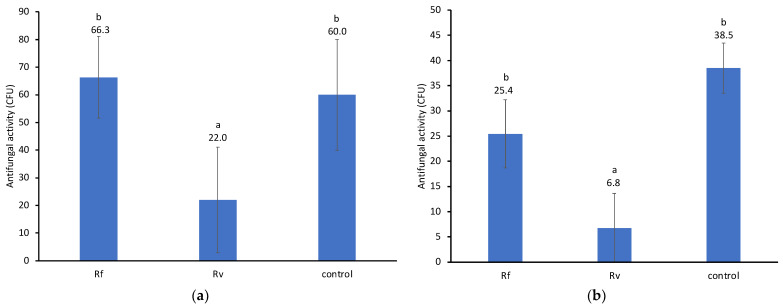
Crude extract antifungal activity against *M. robertsii* (**a**) and *M. brunneum* (**b**). Values above bars represent mean CFUs (±SD), and different letters indicate significant differences (Bonferroni adjusted). Rf corresponds with *R. flavipes* and Rv with *R. virginicus*. The analysis used two replicates from 5 colonies from each termite species, 6 replicates for the Rf control, and 2 replicates for the Rv control.

The two species of fungi were analyzed separately (the viability of conidia on PDA differs between the two species). For *M. robertsii*, mean colony-forming units (CFUs) among fungal and control treatments were significantly different (F = 18.08, df = 2, *p <* 0.001) (Figure 5a). Crude extracts of *R. virginicus* but not *R. flavipes* workers showed significant antifungal activity against *M. robertsii* compared to the control (adjusted *p <* 0.001 and *p =* 1.000, respectively). The antifungal activity of *R. virginicus* extracts against *M. robertsii* was significantly greater than the antifungal activity of *R. flavipes* extracts against *M. robertsii* (adjusted *p <* 0.001). For *M. brunneum*, mean CFUs (Figure 5b) among fungal and control treatments were significantly different (F = 27.26, df = 2, *p <* 0.001). Crude extracts of *R. virginicus* but not *R. flavipes* showed significant antifungal activity against *M. brunneum* compared to the control (adjusted *p <* 0.001 and *p =* 0.072, respectively). The antifungal activity of *R. virginicus* extracts against *M. brunneum* was significantly greater than the antifungal activity of *R. flavipes* against *M. brunneum* (adjusted *p <* 0.001).

### 3.4. Allogrooming among Challenged Pairs

**Figure 6:**
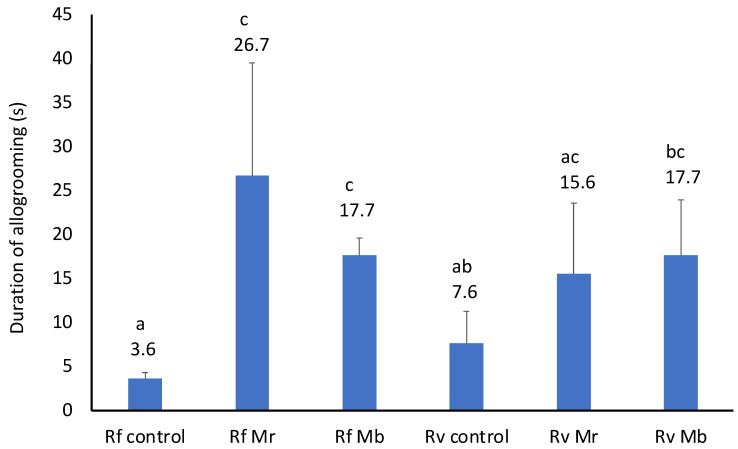
Duration of allogrooming per interaction (n = 161 allogrooming events). Values above bars represent mean duration of allogrooming interactions (±SD), and distinct letter combinations indicate significant differences (Bonferroni adjusted). Rf corresponds with *R. flavipes*, Rv with *R. virginicus*, Mr with *M. robertsii*, and Mb with *M. brunneum*.

The frequency of allogrooming bouts decreased significantly with fungal challenges (challenge mean = 3.55, control mean = 8.80, t = −4.461, *p <* 0.001). However, the mean duration of allogrooming increased significantly with fungal challenges (challenge mean = 19.01 s, control mean = 5.62 s, t = 4.876, *p <* 0.001). The duration of allogrooming bouts for each interaction between termite pairs differed significantly across treatments and controls (F = 7.166, df = 5, *p <* 0.001) (Figure 6). The allogrooming duration per event was significantly elevated in *R. flavipes* pairs treated with *M. robertsii* (adjusted *p <* 0.001) or *M. brunneum* (adjusted *p =* 0.037) relative to controls but not in *R. virginicus* pairs treated with *M. robertsii* (adjusted *p =* 1.000) or *M. brunneum* (adjusted *p =* 1.000) relative to controls. 

## 4. Discussion

*R. flavipes* relies on social mechanisms of defense against local fungal pathogens to a greater extent than *R. virginicus*. For *R. flavipes* workers challenged with either *Metarhizium* species, there was a strong negative correlation between the time two termites spent together and their probability of death relative to challenged single termites. The hazard ratio of death relative to challenged single termites was halved in approximately two hours (Figure 3). Early social interactions therefore appear to be critical for survival when *R. flavipes* workers encounter low concentrations of *Metarhizium* conidia. In contrast, *R. virginicus* workers showed a weak correlation between the time two termites spent together and their probability of death. Also, in contrast to *R. virginicus*, *R. flavipes* worker pairs significantly increased the duration of allogrooming bouts after a fungal challenge (Figure 6). Detection of conidia on a nestmates’ cuticle is likely to have led to this increase. More effective removal of conidia from cuticular surfaces by increasing the duration of allogrooming bouts appears to account for *R. flavipes’* superior social immunity.

Our results also indicate that *R. virginicus* relies on individual innate mechanisms of defense against local fungal pathogens to a greater extent than *R. flavipes*. The mortality of single *R. flavipes* workers challenged with either of the *Metarhizium* species was significantly higher than the mortality of *R. virginicus* workers (Figure 4). The antifungal activity of *R. virginicus* worker cuticular washes against *Metarhizium* conidia has previously been shown to be significantly stronger than the activity of *R. flavipes* workers [20]. The antifungal activity of crude extracts of whole termites was also stronger in *R. virginicus* than *R. flavipes* (Figure 5). These extracts include compounds from intestinal symbionts, which, together with termite antifungal compounds, may also contribute to eliminating ingested conidia [26]. The variation in hazard ratios of death and their weak correlation with separation times in *R. virginicus* workers is consistent with a greater dependence on innate immune defense mechanisms that vary among individuals rather than social immune mechanisms.

*R. flavipes* also showed a greater dependence on social immunity when groups of 12 workers were briefly exposed to high concentrations of *M. robertsii* conidia. Immediately after exposure to *M. robertsii* conidia, both termite species showed elevated LOMs. This alarm activity was significantly higher in *R. flavipes* than *R. virginicus* (Figure 2), and the resistance to fatal infections was also significantly higher in *R. flavipes* than *R. virginicus* (Figure 1). In contrast, there was an elevated but weak alarm response to *M. brunneum* conidia. Consistent with this result, it has previously been shown that elevated alarm in *R. flavipes* in response to the conidia of different *Metarhizium* strains and species is correlated with elevated allogrooming and survival [23]. *R. flavipes* workers may have a lower threshold than *R. virginicus* workers for recognizing and responding collectively and effectively to cuticular contamination with *M. robertsii* conidia.

*M. robertsii*, which is the most prevalent *Metarhizium* species in our study site [24], may represent a threat to *R. flavipes* that requires a strong social immune response because this termite has relatively weak innate immune defenses against it. The effectiveness of antifungal compounds associated with salivary gland secretions is likely to be essential for the individual defenses observed in this study [20]. These compounds are present on the surface of the workers prior to fungal challenges as they are expressed constitutively and are constantly disseminated by basal levels of allogrooming, which therefore integrates individual and social defenses. This gestalt likely includes the small defensin-like antifungal peptide termicin, which is secreted into the salivary gland and is critically important for defense against fatal infections by *Metarhizium* [27].

A study of *R. flavipes* and *R. virginicus* from the same collection site in Maryland used in this study revealed that termicins recently faced a selective sweep that resulted in a reduction in nucleotide polymorphism, and this reduction was greater in *R. flavipes* than *R. virginicus* [28]. The sweep may have been, and possibly continues to be, more severe for *R. flavipes* because *R. virginicus* has more effective innate immune mechanisms that reduced its reliance on termicins for defense against a recent fungal epidemic. Alternatively, adaptive constraints on termicin evolution (lack of genetic variation) may have left *R. flavipes* with weaker weapons in the face of a novel fungal epidemic. Constraint on adaptive change in termicins under strong selective pressure is also evident in Australian *Nasutitermes* [29]. The stochastic process of gene duplication appears to have released this constraint in different nasute lineages, which resulted in the repeated evolution of two termicins with different molecular charges. This may have benefitted antifungal defense because two termicins with different charge properties restrict opportunities for the evolution of fungal resistance to them. 

Termites exhibiting alarm (LOMs) after exposure to a high dose of conidia were less likely to die over two weeks than individuals showing no alarm. LOMs are positively correlated with *R. flavipes* worker aggregation and allogrooming [23], which suggests that individuals exhibiting alarm in this study solicited more grooming from nestmates. LOMs are rarely reported in research investigating *Metarhizium* infections in termites and have been discounted as playing a role in signaling pathogen exposure [30]. However, they may be missed because they do not occur in isolated individuals, are not substantially elicited by certain strains and species of *Metarhizium*, take approximately 3 min after a challenge before they escalate, and subside after approximately 30 min [22,23]. Our results suggest that LOMs communicate an emergency alert after exposure to a high dose of conidia, which are detected while allogrooming. However, LOMs are not necessary for a proactive behavioral response since the duration of allogrooming increased after a low-dose exposure when LOMs were negligible.

## 5. Conclusions

LOMs appear to be part of an emergency response to heavy cuticular contamination that uses networked communication to quickly escalate the removal and destruction of most conidia before they have a chance to firmly attach and germinate, which takes approximately 12 h [31]. This emergency response is likely to be energetically expensive. Enhanced social immunity in *R. flavipes* in response to *M. robertsii* may be an adaptive tradeoff that *R. virginicus* does not have to make to the same extent because it has more effective innate immune mechanisms. When faced with low levels of pathogen exposure that foraging workers must routinely withstand, allogrooming also appears to compensate for weak innate immune mechanisms. 

## Data Availability

Data available in a publicly accessible repository. The data presented in this study are openly available in Zenodo at: https://doi.org/10.5281/zenodo.7551891 (accessed on 19 January 2023).
